# Factors Associated with Intubation Time and ICU Stay After CABG

**DOI:** 10.5935/1678-9741.20150074

**Published:** 2015

**Authors:** Suzanny Flegler, Flavia Marini Paro

**Affiliations:** 1 Universidade Federal do Espírito Santo (UFES), Vitória, ES, Brazil

**Keywords:** Myocardial Revascularization, Extracorporeal Circulation, Respiration, Artificial, Intubation, Risk Factors, Epidemiology

## Abstract

**OBJECTIVE:**

The aim of this study was to identify factors associated with intubation time and intensive care unit stay after coronary artery bypass grafting with cardiopulmonary bypass.

**METHODS:**

This was a retrospective study, whose data collection was performed in the hospital charts of 160 patients over 18 years, who underwent surgery from September 2009 to July of 2013 in a hospital in the state of Espirito Santo, Brazil.

**RESULTS:**

The mean age of the subjects was 61.44±8.93 years old and 68.8% were male. Subjects had a mean of 5.17±8.42 days of intensive care unit stay and mean intubation time of 10.99±8.41 hours. We observed statistically significant positive correlation between the following variables: patients' age and intubation time; patients' age and intensive care unit stay; intubation time and intensive care unit stay.

**CONCLUSION:**

In conclusion, the study showed that older patients had longer intubation time and increased intensive care unit stay. Furthermore, patients with longer intubation time had increased intensive care unit stay.

**Table:** 

**Abbreviations, acronyms & symbols**	
AMI	= Acute myocardial infarction
CABG	= Coronary artery bypass grafting
CPB	= Cardiopulmonary bypass
DM	= Diabetes mellitus
ICU	= Intensive care unit
IPO	= Immediate postoperative
IT	= Intubation time
MV	= Mechanical ventilation
NIV	= Noninvasive ventilation
NYHA	= New York Heart Association
PO1	= First postoperative day
PO2	= Second postoperative day
PO3	= Third postoperative day
PO4	= Fourth postoperative day
PO5	= Fifth postoperative day
PO6	= Sixth postoperative day
RD	= Renal dysfunction
SD	= Standard deviation
SPSS	= Statistical Package for Social Sciences software

## INTRODUCTION

Patients referred for coronary artery bypass graft (CABG) frequently have one or more comorbidities associated with coronary heart disease, as hypertension, diabetes mellitus (DM), peripheral vascular disease, cerebrovascular disease, renal dysfunction (RD) and class II heart failure or above according to New York Heart Association (NYHA)^[1]^. Some of these comorbidities have been associated with prolonged mechanical ventilation (MV), and a higher risk of re-intubation in the postoperative period after CABG^[2]^.

A recent study reported that postoperative total MV time was an independent risk factor for re-intubation following CABG^[3]^. The occurrence of complications is also increased in older patients undergoing CABG^[1,4]^.

Prolonged MV after CABG is closely linked to increased morbidity and mortality of patients, and longer stays both in hospital and in intensive care unit (ICU)^[2,5,6]^, which generates more costs for the service^[5,6]^. It is important to study the predictors of prolonged MV and ICU stay in these patients because this knowledge may contribute to optimise the management of the most appropriate care for CABG patients.

The aim of this study was to identify factors associated with intubation time (IT) and ICU stay after CABG with cardiopulmonary bypass.

## METHODS

This is a retrospective study composed of patients undergoing CABG, from September 2009 to July 2013 in a hospital in the state of Espírito Santo, Brazil.

Data collection was conducted from October 2013 to February 2014 on hospital charts of patients. Inclusion criteria were all patients who underwent isolated CABG with cardiopulmonary bypass (CPB) use from September 2009 to July 2013, over 18 years, of both genders. Patients who underwent concomitant CABG with valve surgery or other surgeries were not included in accordance with the inclusion criteria. We excluded patients who died and those whose charts did not have enough data for the research. Of the total of 172 charts, 160 were included in the sample. Three were excluded because the patient died in the first days after surgery and other nine did not contain sufficient data.

Patient charts were carefully reviewed and data collected were: chart record number, age, gender, admission date, discharge date, smoking history, preoperative hypertension, preoperative DM, preoperative dyslipidemia, preoperative acute myocardial infarction (AMI), date and time of surgery, number of coronary grafts, length of CPB, date and time of entry in the ICU, date and time of extubation, IT (time in hours from intubation in the surgical room to extubation), re-intubations (yes or not, date and time), date and time of the second extubation, use of noninvasive ventilation (NIV) (day and time in minutes), date and time of discharge in the ICU, length of ICU stay (in days), and number of physical therapy attendances per day on each of the following days after the surgery: immediate postoperative (IPO), first postoperative day (PO1), second postoperative day (PO2), third postoperative day (PO3), fourth postoperative day (PO4), fifth postoperative day (PO5), and sixth postoperative day (PO6).

We observed all ethical aspects covered in the Brazilian standards (Resolution 466/2012-CNS) and international standards (Helsinki Declaration/2008) for research on human beings. The project was approved by the Universidade Federal do Espírito Santo Research Ethics Committee under number 294/10.

Statistical analysis was performed using the Statistical Package for Social Sciences software (SPSS) 22 (SPSS Inc.). Categorical variables were expressed as percentages and continuous variables as mean ± standard deviation (SD). Initially, we used the Kolmogorov-Smirnov test to evaluate the normality of the variables. Due to the breakdown of normality hypothesis in all cases, Spearman correlation coefficient (non-parametric) was used to check the correlation between variables. For comparison between groups we used the non-parametric Mann-Whitney test.

## RESULTS

Study population had a mean age of 61.44±8.93 years old and 68.8% (n=110) were male. The average time of CPB was 58.85±21.89 minutes, and the mean number of grafts per surgery were 2.79±0.88. The mean IT was 10.99±8.41 hours. The mean duration of ICU stay was 5.17±8.42 days. These intraoperative and postoperative characteristics of patients are detailed in Table 1.

**Table 1:** Intraoperative and postoperative characteristics of patients who underwent isolated CABG with CPB.

Variables	n	Lower	Upper	Mean	SD
Age	160	37	80	61.44	8.93
Number of grafts	155	1	7	2.79	0.88
Time of CPB (minutes)	152	23	146	58.85	21.89
ICU stay (days)	142	1	95	5.17	8.42
IT (hours)	105	1.25	52.75	10.99	8.41

CABG=coronary artery bypass grafting; CPB=cardiopulmonary bypass; ICU=intensive care unit; IT=intubation time; SD=standard deviation

We observed significant positive correlation between patients' age and IT; between patients' age and ICU stay; and between IT and ICU stay. Number of grafts and time of CPB did not have significant correlation with IT or ICU stay (Table 2).

**Table 2:** Correlation between IT or ICU stay and continuous variables.

Variables	Spearman correlation coefficient	
	ICU stay (days)	IT (hours)
IT (hours)	0.222*	
Age	0.296*	0.279*
Number of grafts	0.092	0.040
Time of CPB	0.052	0.025

IT=intubation time; ICU=intensive care unit; CPB=cardiopulmonary bypass;

* Statistically significant

There was no significant relationship between ICU stay and any of categorical variables gender, smoking, hypertension, DM, dyslipidemia and previous AMI (Table 3). Similarly, statistically significant relationships between these categorical variables and IT weren't observed (Table 3). The prevalence of these categorical variables observed in the study population was: 68.8% had hypertension; 38.8% had DM (not specified whether type I or II); 38.1% had dyslipidemia, 39.4% were smokers, and 23.1% had previous AMI.

**Table 3:** Relationship between patients' preoperative characteristics, ICU stay and IT.

Variables	Category	ICU stay			IT		
		Mean	SD	P-value	Mean	SD	P-value*
Gender	Male	5.55	9.98	0.731	12.29	9.30	0.051
	Female	4.36	2.96		8.51	5.72	
Smoking	Yes	6.11	12.78	0.724	11.66	10.42	0.772
	No	4.56	3.32		10.53	6.74	
Hypertension	Yes	4.57	3.87	0.913	11.02	8.76	0.978
	No	6.47	13.86		10.92	7.73	
DM	Yes	5.09	4.78	0.767	11.53	8.79	0.242
	No	5.22	10.05		10.65	8.22	
	Yes	4.52	4.10	0.154	13.26	10.83	0.113
Dyslipidemia	No	5.62	10.41		9.60	6.19	
	Yes	4.28	3.35	0.273	8.72	5.85	0.170
Previous AMI	No	5.43	9.39		11.74	9.01	

ICU=intensive care unit; IT=intubation time; DM=diabetes mellitus; AMI=acute myocardial infarction; SD=standard deviation;

* 5Mann-Whitney test

Eight (5%) patients required re-intubation. Only 20 (12.5%) patients underwent some type of NIV during the postoperative period.

Regarding the physiotherapy care received by patients, on the PO1, only 3.1% of patients received physiotherapy care and each of them received only one attendance a day; on the PO1 16.3% of patients received 1 attendance a day and 2.5% received 2 attendances a day; on the PO2 only 5.6% of patients received 1 attendance a day. During PO3, PO4, PO5 e PO6 only 2.5% of patients received 1 attendance a day. Due to the low number of patients who received physiotherapy care, it was not possible to apply statistical tests to relate this variable with the study outcomes.

## DISCUSSION

The average age of patients was similar to other studies in recent years^[1,3,5-7]^, which is in line with the current trend of increasing age of patients undergoing CABG^[1]^.

The major findings of the present study were that patients' age had a significant positive correlation with TI and ICU stay. In addition, TI had a significant positive correlation with ICU stay.

Piotto et al.^[8]^ also reported that age was a predictor of prolonged MV and increased hospital stay. Another study also identified age as an independent predictor of prolonged MV^[9]^. Due to the increasing age of patients undergoing myocardial revascularization in last decades^[1]^, it is important to minimize the risk of prolonged TI and ICU stay in this segment of the population, since prolonged intubation results in significant acute and midterm morbidity and mortality^[2]^.

Regarding the positive correlation between IT and ICU found in this study, Cislaghi et al.^[5]^ reported that prolonged MV is associated with higher length of stay in ICU and total hospitalization time. Similarly, Akdur et al.^[10]^ showed increase in ICU stay for patients who spent more than 24 hours on MV.

In our results, the number of grafts did not have significant correlation with IT or ICU stay, and we found a mean of 2.79±0.88 grafts. This number is smaller than the average number of grafts reported by some authors^[1,2,11,12]^, but our average of grafts was similar to the mean found in another study that also did not observe relationship between number of grafts and length of MV or ICO stay^[10]^.

Guizilini et al.^[13]^ compared CABG postoperative period with and without CPB, and concluded that there are impairments of lung function in both; however, this is significantly higher in patients operated with CPB. Most patients undergoing CPB suffer pulmonary dysfunction^[11,13-15]^. A recent study concluded that compared with CABG with CPB, CABG without CPB reduced postoperative respiratory and renal morbidity, and shortened ICU and hospital stay in high-risk patients^[12]^.

In the present study, the mean time of CPB was 58.85±21.89 minutes. The study by Romanini et al.^[16]^ showed average time of CPD similar to this. Other studies presented higher time of CPB^[5-6,12,17]^, and a recent epidemiological study showed smaller mean time of CPD (46.7±22.9 min) for patients underwent CABG^[7]^.

Akdur et al.^[10]^ evaluated the length of MV on CABG postoperative period and observed that MV was longer in patients undergoing longer duration of CPB. The average time of CPB observed in our study (58.85±21.89 minutes) was smaller than the average times of both groups in that study and perhaps this might explain why we did not find correlation between time of CPB and IT.

CPB time longer than 91 minutes was shown to be an independent predictor of prolonged MV^[5]^. A recent publication^[6]^ concluded that postoperative prolonged MV was associated with longer perfusion times in CABG patients, and showed that every 1-minute increase over 82.5 minutes of CPB time increased risk of delayed extubation by 3.5%. Regarding the observed differences in our results when they are compared with the two studies mentioned above, it is important to note that the average CPB time observed in our study was very shorter than average time of those studies and very few patients in our study had more than 91 minutes of CPB time. Furthermore, in the second study^[6]^, the patient population was comprised of a heterogeneous group of patients who had concomitant procedures along with CABG, such as valve procedures, features that may also explain at least in part the differences found.

Hypertension^[2,6,8]^, DM^[2,6,8]^ and dyslipidemia^[6,8]^ have not been associated with a higher intubation time in patients undergoing CABG, which was also observed in our results.

In several studies, smoking has not been related with increased IT in the postoperative of CABG^[2,6,8,10]^, which corroborates our results. However, a recent study accessed a total of 3730 patients undergoing isolated CABG and reported that the smoking group had significantly longer duration of MV^[18]^.

In this study, the prevalence of previous AMI was 23.1%. An epidemiological study conducted in a Brazilian institution showed a prevalence of 46.9% for previous among AMI 3010 patients undergoing CABG^[7]^.

Our data showed no relation between previous AMI and IT or ICU stay, which corroborates another study^[6]^. However, the mentioned study also assessed previous left ventricular dysfunction, ejection fraction and NYHA class, and showed that postoperative MV had no association with previous left ventricular dysfunction and ejection fraction, but they concluded that postoperative prolonged MV was associated with advanced NYHA class^[6]^. Légaré et al.^[9]^ reported that ejection fraction < 50% is a preoperative independent predictors of prolonged ventilation. In addition, Sá et al.^[19]^ showed that ejection fraction < 50% is a risk factor for low cardiac output syndrome after CABG, and they postulated that patients with low cardiac output after CABG present longer ICU and hospital length of stay. One limitation of our study, which was retrospective, was our inability to assess the information about preoperative ejection fraction, left ventricular dysfunction, heart failure, and NYHA class, since this information was not included in many patients' charts, and even it was present, it was not often written in a form of standardized classification for all patients.

Intraoperative transfusions are independent predictors of prolonged MV^[5]^. It has been reported that limiting intraoperative and postoperative blood product transfusion decreases adverse postoperative events such as prolonged MV^[20]^. The mortality risk is directly proportional to the number of packed red blood cells transfused in CABG^[21]^. However, in the present study, information about blood product transfusions was not collected.

Akdur et al.^[10]^ reported that 2.61% of patients undergoing CABG required re-intubation. In the present study we observed that 5% of patients were re-intubated. It is very well established in the literature that the use of NIV in the postoperative period of cardiac surgery improves oxygenation^[22,23]^, reduces rate of re-intubation^[23]^, reduces recovery time and median duration of hospital stay^[24]^. In this study, only 20 (12.5%) patients received NIV in the postoperative period, and among patients who were re-intubated, only 2 (25%) had received NIV before reintubation.

When we analyzed the average of ICU stay, we found a mean of 5.17±8.42 days. Most authors who had studied this variable found a lower ICU stay^[3-6,12,18,24,25]^ some of them reported almost half of our time or even less^[4-5,18]^.

Most patients in this study received no physiotherapy care during ICU stay, so it was not possible to apply statistical tests to relate this variable with the study outcomes. It occurred because during most of the study period, the ICU of hospital studied had only one single professional physiotherapist for 30 hours per week, with no physical therapists to assist patients during several hours a day, on weekends, at night, or in the rooms after discharge from the ICU. Although it was not possible to apply tests to correlate the variable physiotherapy care with IT and ICU stay, it is important to note that several studies have reported the importance of physiotherapy care in the pre and postoperative period of CABG in preventing complications^[15,22,26,27]^, improvement of hypoxemia^[16,22,28]^, improvement of tidal volume and vital capacity^[29]^, reducing the time of TI^[26,28]^, reducing the hospital stay^[26]^ and prevention of reintubation^[23]^.

In regard to limitations of this study, the main limitation was its retrospective nature, because data were collected from hospital patients' charts that had often incomplete or imprecise information, accounting data loss, which cause considerable possibility of bias.

## CONCLUSION

In conclusion, the study showed that older patients had higher IT and increased ICU stay. Furthermore, patients with longer IT had higher ICU stay.

**Table:** 

**Authors' roles & responsibilities**	
SF	Study conception and design; implementation of projects/experiments; analysis and/or interpretation of data; statistical analysis; manuscript writing or critical review of its contents; final approval of the manuscript
FMP	Study conception and design; analysis/interpretation of data; statistical analysis; manuscript writing or critical review of its contents; final approval of the manuscript
